# A case report: First presentation of diabetes mellitus type 1 with severe hyperosmolar hyperglycemic state in a 35‐month‐old girl

**DOI:** 10.1002/ccr3.4984

**Published:** 2021-11-06

**Authors:** Amir Saeed, Homa Ilkhanipoor

**Affiliations:** ^1^ Division of Pediatric Intensive Care Department of Pediatrics Shiraz University of Medical Sciences Shiraz Iran; ^2^ Division of Pediatric Metabolism and Endocrinology Department of Pediatrics Shiraz University of Medical Sciences Shiraz Iran

**Keywords:** case report, diabetes mellitus, hyperglycemic hyperosmolar syndrome (HHS), pediatric patients, rhabdomyolysis, thrombosis

## Abstract

Hyperglycemic hyperosmolar syndrome (HHS) is a rare complication of diabetes mellitus among pediatric patients. Since its treatment differs from diabetic ketoacidosis (DKA), hence, pediatricians should be aware of its diagnosis and management.

## Graphical abstract

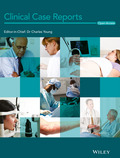

## 1 BACKGROUND

Hyperglycemic hyperosmolar syndrome (HHS), which is characterized by increased serum glucose concentrations and hyperosmolality with low or absence of ketonemia or ketonuria, has been rarely reported in children. Herein, we report a 35‐month‐old girl, who was newly diagnosed with diabetes mellitus type1 (T1DM), with presentation of HHS that developed some complications.

Hyperosmolar hyperglycemic state (HHS), a rare diabetic hyperglycemic emergency, is most often observed in adult patients, but seldom seen in pediatric patients. Nevertheless, it can present in younger adults and teenagers as the first presentation of diabetes mellitus type 2 (T2DM).^1^

HHS is diagnosed *by* the following criteria: plasma glucose more than 600 mg/dl, venous pH > 7.25, serum bicarbonate >15 mmol/L, small amount of ketonuria or its absence, effective serum osmolality >320 mOsm/kg, and obtundation, combativeness, or seizures (in approximately 50% of all cases).^1^, ^2^

The incidence of HHS has merely been reported in 0.8–2% of all pediatric patients,^3^, ^4^ but it has a higher mortality rate in children compared to DKA (~10–35%).^5^

Among the precipitating factors for HHS, the main one is infection which has to be diagnosed and treated immediately.

DKA mostly develops within hours of its onset, and the main presentations are as follows: hyperventilation, vomiting, and abdominal pain which force the parents to take their children to a physician. On the contrary, HHS develops over several days and presents itself later on. In this case, patients have polyuria and polydipsia for a longer period; hence, it might not be recognizable, and ultimately present itself with severe dehydration and electrolyte disturbance. It should be noted that the degree of dehydration, electrolyte, and metabolic disturbances are more severe.

Although in HHS there is substantial loss of electrolyte, and volume, the signs of dehydration are not recognizable, due to either obesity or hypertonicity. Hence, clinical assessment of dehydration becomes more difficult. Moreover, treatment of children with HHS differs from DKA; as in patients with HHS, intravascular volume should be replaced more and faster compared to DKA in order to avoid vascular collapse.

The recommendations in the treatment of HHS in pediatric patients are based on adult experiences. The first step is fluid therapy in order to expand intra‐ and extravascular fluid to preserve renal perfusion; hence, the rate of hydration becomes much faster than DKA.^1^, ^2^

Nonetheless, there are some HHS’ related complications that can be life‐threatening, such as vascular complications (eg, myocardial infarction, stroke, and peripheral arterial thrombosis), central nervous system complications (eg, seizures, cerebral edema, and central pontine myelinolysis (CPM)), which are uncommon, but described as HHS complications.^1^, ^2^

In this case report, a 3‐year‐old girl is presented with HHS who developed some complications.

## 2 CASE PRESENTATION

A previously healthy 35‐month‐old girl was brought to the emergency room of the Namazi hospital, Shiraz, Iran, due to reduced level of consciousness. She was well up to five days prior to her admission, after that she presented with dysuria and loss of appetite, and then developed polyuria, polydipsia, and weight loss (14 kg → 11 kg). There was no history of DM in her family.

On arrival, her height was measured 92 cm (25th−50th) percentile), she weighed 11 kg (5th–10th) percentile), and her Body Mass Index (BMI) (BMI‐for‐age) was at the 72nd percentile. Her vital signs were as follows: temperature: 38°C, heart rate (HR): 160, blood pressure (BP): 95/50, and her Glasgow coma scale (GCS) was 11/15. During her physical examination, capillary refilling time was more than 3 s with weak pulses. When checked with a capillary blood glucose meter, her glucose level was too high, so a serum glucose test was performed. Moreover, her first VBG was as follows: pH 7.26, PCO_2_: 32, PO_2_: 39, HCO_3_: 15.8, and BE: −12.6. Due to the decreased level of consciousness, brain CT scan was performed in which brain edema was reported.

As the first line of treatment, she was hydrated with normal saline (10 cc per kg), then the laboratory report revealed; blood sugar: 1124 mg/dl, BUN: 71 mg/dl, creatinine: 1.9 mg/dl, Na: 170 mEq/L (corrected Na: 186), and K: 5.1 mEq/L (effective osmolality was 402). Additionally, urine analysis was SG: 1.010, GLU: 3+, Ketone: trace. VBG after first hydration was as follows: pH: 7.27, PCO_2_: 34.8, and HCO_3_: 15.8. Based on the laboratory report; HHS was confirmed as diagnosis; hence, she was transferred to the Pediatric Intensive Care Unit (PICU).

On arrival at PICU, she developed generalized convulsion, and her GCS declined to less than 8; so she was intubated.

She was hydrated with normal saline again; then, the intravenous fluid was administered with 15 percent deficit and maintenance of fluid in addition to urine output replacement. Due to brain edema, deficit was given over 72 h, but according to urine output and serum sodium level, the amount of deficit increased up to 18 percent, and the sodium content of IV fluid decreased.

The first sodium level reported in PICU was 185 mEq/L, but it gradually decreased as hydration continued, and the amount of fluid increased (with the target level of 10 mEq/L decrease per day); then, it became stable around 145 mEq/L over the next five days. Our primary goal in her treatment was to correct sodium level maximum 10 mEq/L per day, to decrease osmolality maximum 3–5 mosm/L/h, and to reduce blood sugar maximum 75 mg/dl per hour.

On the 3rd day, HHS was resolved, but the patient became febrile, and the amount of endotracheal tube (ETT) secretion increased. The culture of ETT secretions revealed candida non‐albicans and pseudomonas aeruginosa; hence, antibiotic was prescribed. Subsequently, the patient developed hypotension, so inotrope was initiated. On the same day, her blood creatine phosphokinase (CPK) increased to 6400 IU/L, and it reached 13,400 on the fifth day. Then, she developed hemoglobinuria; thereafter, hydration continued until the CPK level returned normal level on the 18th day (Table 1).

**TABLE 1:** Laboratory findings

Tests		Results (day 1)	Day 4	Day 14
Sodium (135–145 mEq/L)		170	154	138
Potassium (3.5–5.1 mEq/L)		5.1	3.4	4.7
Chloride		95	128	105
Phosphorus (3.4–4.5 mg/dl)		3.1	1.2	4
Serum glucose (mg/dl)		1124	220	180
Blood urea nitrogen		71	18	9
Creatinine		1.9	0.6	0.4
Venous blood gas	pH	7.26	7.40	
	HCO_3_	15.8	24	
Effective osmolality (275–295 mOsm/kg)		402		
Urine analysis (ketone)		+/−	Negative	Negative
CPK^a^		212	6400	256
Magnesium (1.7–2.2 mg/dl)		3.1	1.5	2.2
Calcium (8.5–10.5 mg/dl)		10	8.7	10
CRP^b^ (<5)		4	>150	3

^a^ CPK: Creatine phosphokinase.

^b^ CRP: C‐reactive protein.

Although enoxaparin was initiated for deep venous thrombosis (DVT) prophylaxis, she presented with the left forearm and hand swelling, on the 6th day. Doppler ultrasonography revealed thrombosis of the distal part of the brachial artery, so the therapeutic dose was initiated. Consequently, her conditions improved after 2 days, and a week later, Doppler sonography was normal.

On the 9th day, she was weaned off the ventilator, and on the 21st day, the patient was discharged from hospital without any sequela.

## 3 DISCUSSION AND CONCLUSIONS

Hyperglycemic hyperosmolar syndrome (HHS) is a rare presentation of DM in pediatric patients, especially as the first presentation of T1DM, with a high mortality rate. HHS is not easy to diagnose according to physical examination, patient's past medical history or even with availability of laboratory data. However, physicians do not need to be too concerned of HHS in young children, especially if the patient is not obese.^5^

Contrary to the frequent symptoms of DKA, such as vomiting, abdominal pain, and drowsiness that force parents to refer to a hospital, the gradual increase of HHS symptoms can cause delayed referral which can ultimately result in severe dehydration and electrolyte imbalance. As a result, proper diagnosis can lead to appropriate management.

HHS generally occurs among obese people and in T2DM,^6^ but there are some rare reports on HHS in non‐obese patients and HHS in T1DM.^7^ It should be noted that our patient had a normal BMI and T1DM.

Fluid deficits in HHS patients are frequently 12–15% that has to be corrected gradually and uniformly over 24–48 h, but it can be increased up to 20% or more to gradually decline serum sodium and osmolality.^1^, ^2^

At present time, there is no standard therapeutic guideline for HHS in children. Nonetheless, the two most important points in HHS management are fluid replacement, and gradually reduction of serum osmolality and sodium level. Fluid replacement in children with HHS should be carried out more swiftly with more amount of fluid in comparison with children with DKA. To gradually reduction of hypernatremia, we constantly measured her serum sodium level in order to adjust fluid sodium concentration. Due to the presence of brain edema, reaching the aforementioned goals became more difficult and required more attention.

The insulin infusion strategy might differ from insulin infusion rate, which is 0.1 unit/kg/h in patients with DKA, whereas it should be 0.025–0.05 unit/kg/h in patients with HHS.^1^ Our patient was first diagnosed with DKA and treated as DKA in the emergency room; she was hydrated with 10 cc/kg normal saline, and deficit volume was estimated 10%; Insulin treatment was initiated, but after 3 h sodium level increased to 185 mEq/L. According to HHS protocol in PICU, insulin infusion was stopped and the patient hydrated up to 40 cc/kg with normal saline till the patient's hemodynamic became stable. Then, after the initial rehydration, insulin infusion was initiated with 0.03 units per kg per hour, and after 8 h, the sodium content of IV fluid was steadily reduced to 100 mEq/L.

Initially, the degree of dehydration was estimated 15%, but according to serum osmolality, serum sodium, and urine out, the percentage of deficit had increased to 18%.

There are some serious complications in HHS, for example, brain edema, arterial and venous thrombosis, and rhabdomyolysis.^8^ In our patient, the complications were brain edema, rhabdomyolysis, and arterial thrombosis (distal part of brachial artery). The patients with HHS are at risk of venous thrombosis, especially those who are immobile more than 48 h, and for those who central venous catheter is inserted.^1^, ^8^ Although at the beginning of PICU admission we started enoxaparin for prophylaxis, she presented arterial thrombosis.

Altered level of consciousness is commonly seen in adult patients with osmolality more than 330 mOsm/kg, but brain edema rarely occurs in HHS.^1^, ^9^ At the time of admission, our patient's level of consciousness was low, which was due to high osmolality and brain edema (it was diagnosed clinically, and confirmed by brain CT scan). She also developed generalized convulsion; hence, she was intubated. Our goal was to gradually decrease serum osmolality and to administer fluids over a 72‐h period. Based on serum osmolality, urine out, and sodium level, we increased the amount of fluid. Moreover, neuroprotection was started for her (head of bed elevation 30‐degree, mannitol 20%, assuring adequate oxygenation by saturations >90%, avoiding hypercapnia by PaCO_2_ between 34 and 38, and appropriate mean arterial pressure (MAP) to maintain adequate cerebral perfusion pressure in the range of 50–70, and aggressive fever control).^9^

Deficit of potassium, magnesium, and phosphate in HHS is much greater than DKA. In our patient, on the 4th day of admission, serum phosphate decreased to 0.75 mg/dl.

In the previous studies, mortality rate has been reported up to 35% depending on the severity of dehydration, hyperosmolality, and patient's age.^5^ However, by following the aforementioned therapeutic procedures, our patient was cured without any sequelae.

HHS is a rare complication of DM among pediatric patients, but with more complications and poorer outcome. Hence, pediatrician should be well aware of its presentations and signs for a timely diagnosis and treatment.

## CONFLICT OF INTERESTS

The authors declare that they have no competing interests.

## AUTHOR CONTRIBUTIONS

AS designed the study and wrote the manuscript, in addition to data collection as well as submitting the manuscript. HI was the scientific consultant. AS and HI edited the manuscript collectively. Both authors discussed the results and contributed to the final manuscript.

## ETHICAL APPROVAL

This study was approved by the local ethics committee of Shiraz University of Medical sciences with approval ID: IR.sums.med.rec.1398.134. Written informed consent was obtained from patient's parents and delivered to the ethics committee.

## CONSENT

Written informed consent was obtained from the parents of the patient for publication purposes of this case report and any accompanying images. A copy of the written consent is available for review by the Chief Editor of the Journal.

## Data Availability

All data generated or analyzed during this study are included in this published article.
